# Healthy Lifestyle and Time to Depression Onset in Individuals With and Without Incident Stroke: A Longitudinal Analysis Using UK Biobank Data

**DOI:** 10.31083/AP49487

**Published:** 2026-06-28

**Authors:** Chengguang Zou, Guo Li

**Affiliations:** ^1^Department of Neurology, Tongji Hospital, Tongji Medical College, Huazhong University of Science and Technology, 430030 Wuhan, Hubei, China

**Keywords:** depression, healthy lifestyle score, risk factors, stroke, accelerated-failure-time model

## Abstract

**Background:**

Depression and stroke are leading causes of the global health burden. Adherence to a healthy lifestyle can reduce the risk of both conditions. However, the quantitative effect of a healthy lifestyle on the timing of depression onset remains unclear, as does whether the association between lifestyle and depression differs according to the occurrence of stroke. We therefore examined whether the association between a healthy lifestyle and depression differed between individuals who did and did not experience an incident stroke.

**Methods:**

Using data from the UK Biobank, we assessed the associations between lifestyle factors and time to depression using accelerated-failure-time models. Subgroup analyses were conducted to examine whether the association between lifestyle factors and time to depression differed according to the occurrence of incident stroke.

**Results:**

A total of 165,941 participants were included in the study. During follow-up, 5507 individuals developed depression, among whom 103 had previously developed stroke. Participants with 6–7 healthy lifestyle behaviors exhibited an 81% longer time to depression onset than did those with 0–2 healthy lifestyle behaviors after adjusting for covariates. Each one-point increase in the healthy lifestyle score (HLS) was associated with approximately 16.2% prolongation in time to depression. The association between HLS and time to depression onset was observed both in participants with and without incident stroke, although effect estimates were attenuated among those with incident stroke.

**Conclusions:**

The protective association between HLS and delayed depression onset was consistently observed in individuals both with and without incident stroke. Although the association was weaker among those who experienced stroke, lifestyle factors remained strongly associated with depression risk, underscoring the importance of lifestyle modification for mental health prevention irrespective of stroke status.

## Main Points

1. A healthy lifestyle delays depression onset. In this large UK Biobank cohort, a higher healthy lifestyle score (HLS) was associated with a longer time to incident depression (about 16.2% longer time per 1-point HLS increase), showing a clear protective association.

2. Stroke modifies the magnitude, but not the direction, of the association. In subgroup analyses stratified by incident stroke, higher HLS was associated with a longer time to depression onset in both groups, although the strength of the association was attenuated among individuals with incident stroke.

3. Clinical implications: lifestyle modification should be prioritized for mental health prevention, not solely for vascular risk reduction.

## 1. Introduction

Depression represents one of the major public global health challenges, affecting millions of individuals and imposing substantial socioeconomic burdens worldwide. Depression, characterized by persistent sadness and a loss of interest or pleasure in activities, affects over 264 million people globally and is a leading contributor to the global burden of disease [1]. Modifiable lifestyle factors represent one of the key directions for its prevention and management. Various elements, including diet, physical activity, sleep patterns, and alcohol or tobacco use, have been correlated with depressive symptoms in individuals with major depressive disorder (MDD) [2]. Findings from a recent meta-review in lifestyle psychiatry demonstrate with moderate-to-strong evidence that physical activity, diet, smoking, and sleep are key modifiable factors for preventing and treating depression [3]. Recent evidence has further supported the protective role of healthy lifestyle behaviors for mental health. For example, one review paper reported that physical-activity interventions improve depressive symptoms across diverse adult populations, and a recent meta-analysis found higher daily step counts to be associated with a lower risk of depression [4,5].

Results from a meta-analysis of the literature also indicate that healthy lifestyle behaviors may have an additive or multiplicative effect in reducing symptoms of depression [6]. This aligns with prior meta-analytic findings showing that a greater number of healthy lifestyle behaviors is associated with significantly lower odds of depressive symptoms [7]. Multiple underlying pathways may explain how healthy lifestyle behaviors contribute to improved mental health outcomes. For instance, physical activity enhances brain function in regions linked to depression and stress regulation, while also fostering psychosocial resources like self-esteem and social connectedness [8]. A healthy diet, on the other hand, can modulate the gut-brain axis as well as inflammatory and oxidative stress pathways [9]. Conversely, disrupted sleep often exacerbates mental health conditions by impairing psychological and social functioning [10]. Contrary to common perception, smoking may not alleviate but exacerbate mental health symptoms. This worsening is likely due to the mood fluctuations associated with nicotine withdrawal [11].

Furthermore, while extensive evidence links individual lifestyle factors to mental health, research syntheses have largely focused on these independent associations. Recent efforts to analyze interventions targeting multiple behaviors (e.g., combined diet and exercise programs) show these integrated approaches can have a modest positive impact on depression [12,13]. Consequently, adopting these healthy lifestyle behaviors plays an important role in overall mental health prevention, management, and treatment. Nevertheless, there is a scarcity of research specifically investigating the relationship between healthy lifestyle and the time to depression onset. This association remains largely unexplored. Therefore, quantifying the precise time-to-onset benefit of a healthy lifestyle in delaying depression, particularly within contexts of major health events like stroke, is crucial for developing targeted primary prevention strategies.

It is noteworthy that these lifestyle factors not only directly impact mental health but also operate through shared pathways such as cerebrovascular health. Lifestyle factors probably represent a shared etiology for both stroke and depression. For instance, sleep-related breathing disorders (e.g., obstructive sleep apnea) and poor sleep quality have been linked to increased stroke risk and poorer post-stroke recovery, potentially through intermittent hypoxia, systemic inflammation, and impaired cerebral blood flow regulation. These sleep disturbances also commonly co-occur with depressive symptoms, underscoring the interconnected sleep–stroke–mental health pathway [14]. Lee et al. [15] further demonstrated that an unhealthy lifestyle contributes to both elevated stroke risk and more severe depressive symptoms. Stroke itself is also an established risk factor for depression, with approximately 33% of patients developing depressive disorders after a stroke event [16]. The physical and cognitive impairments caused by stroke often markedly reduce quality of life and personal autonomy, which can foster feelings of hopelessness and precipitate depression [17].

We aimed to investigate, using UK Biobank data, whether a healthy lifestyle delays the onset of depression, analyzing this relationship separately in individuals with and without a subsequent stroke to clarify its role across distinct clinical contexts.

## 2. Materials and Methods

### 2.1 Study Sample

UK Biobank is a national prospective cohort study in the United Kingdom that recruited 502,401 participants between 2006 and 2010. Participants attended a baseline assessment at one of 22 evaluation centers across England, Scotland, and Wales. 

Baseline data in the UK Biobank comprise self-reported questionnaires, interview records, health records, physical measurement data, and blood sample data. Further details regarding the questionnaires and interviews are available on the UK Biobank website (https://biobank.ndph.ox.ac.uk/) and in the supplementary documentation (https://www.ukbiobank.ac.uk/wp-content/uploads/2025/01/Main-study-protocol.pdf) published by UK Biobank.

We first excluded participants with stroke, depression or myocardial infarction (MI) at baseline; the remaining individuals were further screened based on the completeness of data related to lifestyle factors (smoking, alcohol intake, diet, physical activity, sleep duration, cognitive activity and social contact).

### 2.2 Healthy Lifestyle Score

We constructed a healthy lifestyle score (HLS) based on seven dimensions: no current smoking, moderate alcohol consumption, a healthy diet, engagement in moderate physical activity at least twice per week, 6–9 h/night of sleep, active participation in cognitive activities, and maintenance of social contacts.

Moderate alcohol consumption was defined as a daily alcohol intake of no more than 16 g for men or 8 g for women.

A healthy diet was determined by the intake levels of the following food categories: fruits, vegetables, whole grains, refined grains, dairy products, vegetable oil, fish, processed meats, red meats and sugar-sweetened beverages. Specifically, the ten components of a healthy diet included increased consumption of fruits, vegetables, whole grains, dairy products, vegetable oil, and fish, as well as the reduced consumption of refined grains, processed meats, red meats and sugar-sweetened beverages. Meeting at least half of the diet components was considered an ideal diet according to the original definition [18]; therefore, with ten components in our study, we defined a healthy diet as meeting ≥5 of 10 components.

Moderate physical activity was defined as at least 150 min/week of moderate-intensity activity or 75 min/week of vigorous-intensity activity.

Cognitive activities included sports/gym activities, pub/social club attendance, religious group participation, adult education classes, or other group activities. Participation in no fewer than three of the aforementioned activity types was considered active engagement in cognitive activities [19].

Social contacts were assessed across two dimensions: feelings of loneliness and frequency of confiding in others. Participants who frequently felt lonely or never confided in others were defined as having no social contact [20].

Each healthy lifestyle item was dichotomized into 0 or 1, where 1 indicated a healthy status and 0 indicated an unhealthy status. The sum of scores for all healthy lifestyle items constituted the HLS, with a range of 0 to 7.

### 2.3 Incident Stroke and Depression Outcomes

For incident stroke, we used algorithmically defined outcomes, including stroke, ischaemic stroke, intracranial hemorrhage, and subarachnoid hemorrhage. Incident depression was ascertained using hospital records containing admission and diagnostic data. Participants with depression were identified as those with a primary or secondary diagnosis of depression in hospital records. All outcomes were defined in accordance with the International Classification of Diseases, 10th Revision (ICD-10) coding system: stroke (I60, I61, I62.9, I63, I64, I67.8, I69.0, and I69.3) and depression (F32 and F33).

### 2.4 Statistical Analysis

Baseline characteristics of the study sample were summarized by HLS category, with categorical variables presented as percentages and continuous variables presented as means and standard deviations (SDs). Differences in baseline characteristics of the study sample across HLS categories (0 to 7) were compared using analysis of variance (ANOVA) or Mann–Whitney U test for continuous variables and Chi-squared tests for categorical variables.

Due to the violation of the proportional hazards assumption, variable-adjusted accelerated-failure-time (AFT) models were applied in our study. The AFT model is a parametric model for survival analysis that directly models survival time; its regression coefficients are interpreted as a time ratio, i.e., the difference in the rate of the outcome occurrence associated with two different values of the exposure variable. We applied the AFT model to calculate the time ratio and 95% confidence intervals (CIs) for the association between HLS and incident depression.

Follow-up duration was calculated as time from baseline assessment to the first occurrence of depression, death, loss to follow-up, or the end of follow-up (19 July 2022, the date of the last hospital admission). Age was used as the time scale. Multiple imputation was used to address missing data (including sociodemographic variables and clinical covariates used in Models 1–3). Five entered datasets were generated under a chained-equations framework, and estimates were pooled using Rubin’s rules. The imputation model included the exposure (HLS), outcomes, and all covariates included in the main analyses. All variables included in the main analyses were incorporated into the imputation model.

Three statistical models were constructed. Model 1 adjusted for sex, age, ethnicity, education level, employment status, Townsend Deprivation Index, and income. Model 2 additionally adjusted for body mass index (BMI), history of diabetes mellitus (DM), hypertension (HTN) and ischaemic heart disease (IHD). Model 3 further incorporated adjustments for cholesterol level and lowdensity lipoprotein (LDL) levels.

Participants holding a college/university degree or professional qualifications (e.g., in nursing or teaching) were classified as having higher education attainment. The history of IHD was determined through either medical record (ICD-10 codes I20–I25) or self-reported diagnosis. DM history was ascertained based on medical records (ICD-10 codes E10-E14), glycated hemoglobin levels ≥6.5%, use of glucose-lowering medications, or self-report. HTN was defined as systolic blood pressure (SBP) ≥140 mmHg or diastolic blood pressure (DBP) ≥90 mmHg, current use of antihypertension agents, documented medical records (ICD-10 codes I10-I13 and I15), or self-report.

Restricted cubic spline models were used to evaluate the relationship between HLS and incident depression, with 4 knots at the 25th, 50th, 75th, and 95th centiles. In the spline models, we adjusted for sex, age, employment, deprivation index, income level, BMI, history of DM, history of IHD, history of HTN, cholesterol level and LDL level.

### 2.5 Subgroup Analysis

We performed subgroup analyses to examine whether the association between HLS and incident depression differed according to the occurrence of incident stroke. Incident stroke was defined as stroke occurring after baseline recruitment and before the onset of depression. Participants were stratified into two subgroups based on whether an incident stroke occurred during follow-up.

Within each subgroup, accelerated-failure-time (AFT) models were fitted to estimate the association between HLS and time to incident depression, with results expressed as time ratios and 95% CIs. All models were adjusted for the same set of covariates as in Model 3 of the main analysis. Statistical analyses were performed using R software (version 4.4.1; R Foundation for Statistical Computing, Vienna, Austria).

## 3. Results

### 3.1 Study Sample

**Fig. 1.**
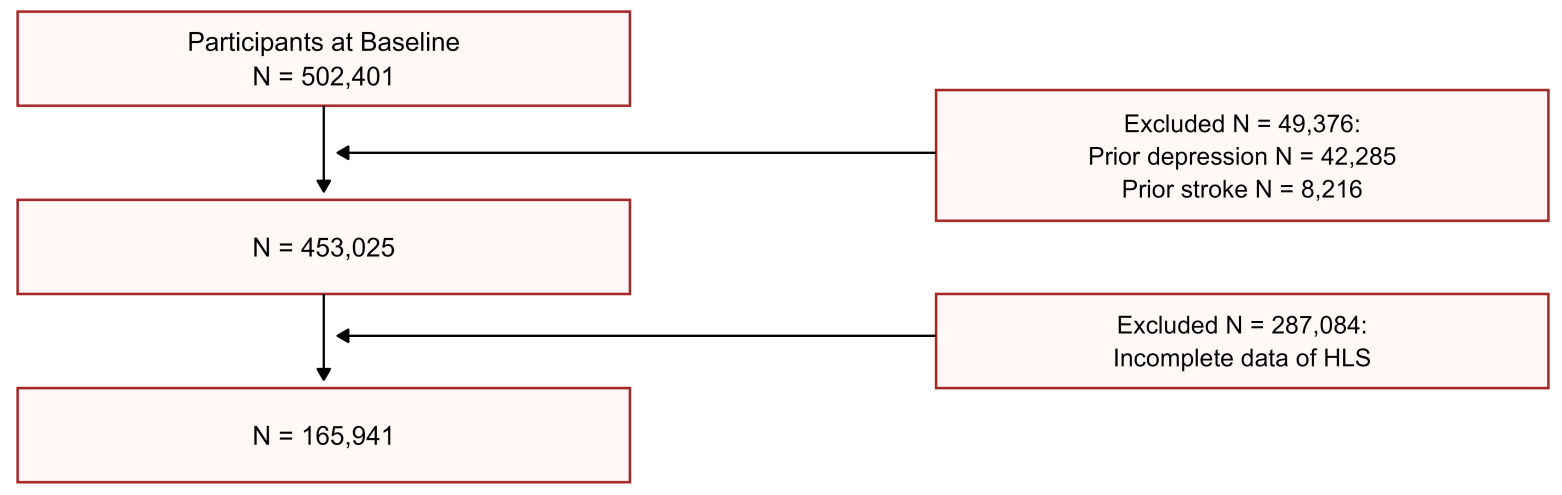
**Flowchart of participants selection**. Of the 42,285 participants with baseline depression and 8216 participants with baseline stroke, 1125 presented with both conditions at baseline.

Participants with a prior history of depression (n = 42,285) or stroke (n = 8216) at baseline, as well as those with missing data on healthy lifestyle factors (n = 287,084), were excluded from the analysis. The final analytical sample consisted of 165,941 individuals (Fig. 1). Among them, 17,485 (10.5%), 37,178 (22.4%), 53,217 (32.1%), 41,220 (24.8%), and 16,841 (10.1%) had HLS values of 0–2, 3, 4, 5 and 6–7, respectively. Participants with higher HLS were more likely to be female, more highly educated, wealthier, have a lower BMI, fewer histories of DM, HTN and IHD, as well as lower levels of LDL and total cholesterol compared to those with low HLS (0-2). Detailed demographic characteristics of the enrolled participants are presented in Table 1.

**Table 1.** **Baseline demographics of the study population according to healthy lifestyle score**.

	HLS						*p* value†
	Total	0–2	3	4	5	6–7	
Number of patients	165,941	17,485	37,178	53,217	41,220	16,841	
Age, years	56.47 ± 8.14	56.12 ± 8.02	56.32 ± 8.08	56.41 ± 8.14	56.56 ± 8.17	57.19 ± 8.28	<0.001
Men	87,703 (52.85)	10,512 (60.12)	21,194 (57.01)	28,044 (52.70)	20,281 (49.20)	7672 (45.56)	<0.001
Ethnicity, White	152,605 (91.96)	15,960 (91.28)	34,126 (91.79)	48,936 (91.96)	38,028 (92.26)	15,555 (92.36)	<0.001
Education, High level	75,888 (45.73)	6856 (39.21)	15,771 (42.42)	24,497 (46.03)	19,984 (48.48)	8780 (52.13)	<0.001
Townsend Deprivation Index	–1.82 ± 2.76	–1.41 ± 3.00	–1.67 ± 2.83	–1.83 ± 2.75	–1.99 ± 2.66	–2.14 ± 2.55	<0.001
Employment, employed	158,516 (95.53)	16,556 (94.69)	35,537 (95.59)	50,858 (95.57)	39,415 (95.62)	16,150 (95.90)	<0.001
Income, less than 18,000	21,129 (12.73)	2739 (15.66)	5033 (13.54)	6374 (11.98)	4914 (11.92)	2069 (12.29)	<0.001
BMI, kg/m^2^	26.67 ± 4.08	27.62 ± 4.29	27.15 ± 4.13	26.63 ± 4.00	26.24 ± 3.99	25.78 ± 3.83	<0.001
History of DM	6285 (3.79)	799 (4.57)	1522 (4.09)	1998 (3.75)	1402 (3.40)	564 (3.35)	<0.001
History of HTN	82,828 (49.91)	9325 (53.33)	19,275 (51.85)	26,427 (49.66)	19,761 (47.94)	8040 (47.74)	<0.001
History of IHD	6393 (3.85)	864 (4.94)	1579 (4.25)	2033 (3.82)	1384 (3.36)	533 (3.16)	<0.001
Cholesterol, mmol/L	5.73 ± 1.10	5.77 ± 1.10	5.76 ± 1.11	5.73 ± 1.09	5.70 ± 1.10	5.70 ± 1.09	<0.001
LDL, mmol/L	3.57 ± 0.84	3.59 ± 0.84	3.58 ± 0.85	3.56 ± 0.83	3.55 ± 0.84	3.56 ± 0.83	<0.001

Data are presented as mean ± SD or n (%). †*p* values for differences in baseline characteristics were estimated by ANOVA or chi-squared test. Abbreviations: HLS, healthy lifestyle score; ANOVA, analysis of variance; BMI, body mass index; DM, diabetes mellitus; HTN, hypertension; IHD, ischaemic heart disease; LDL, low density lipoprotein.

### 3.2 Association Between HLS and Time to Depression

**Fig. 2.**
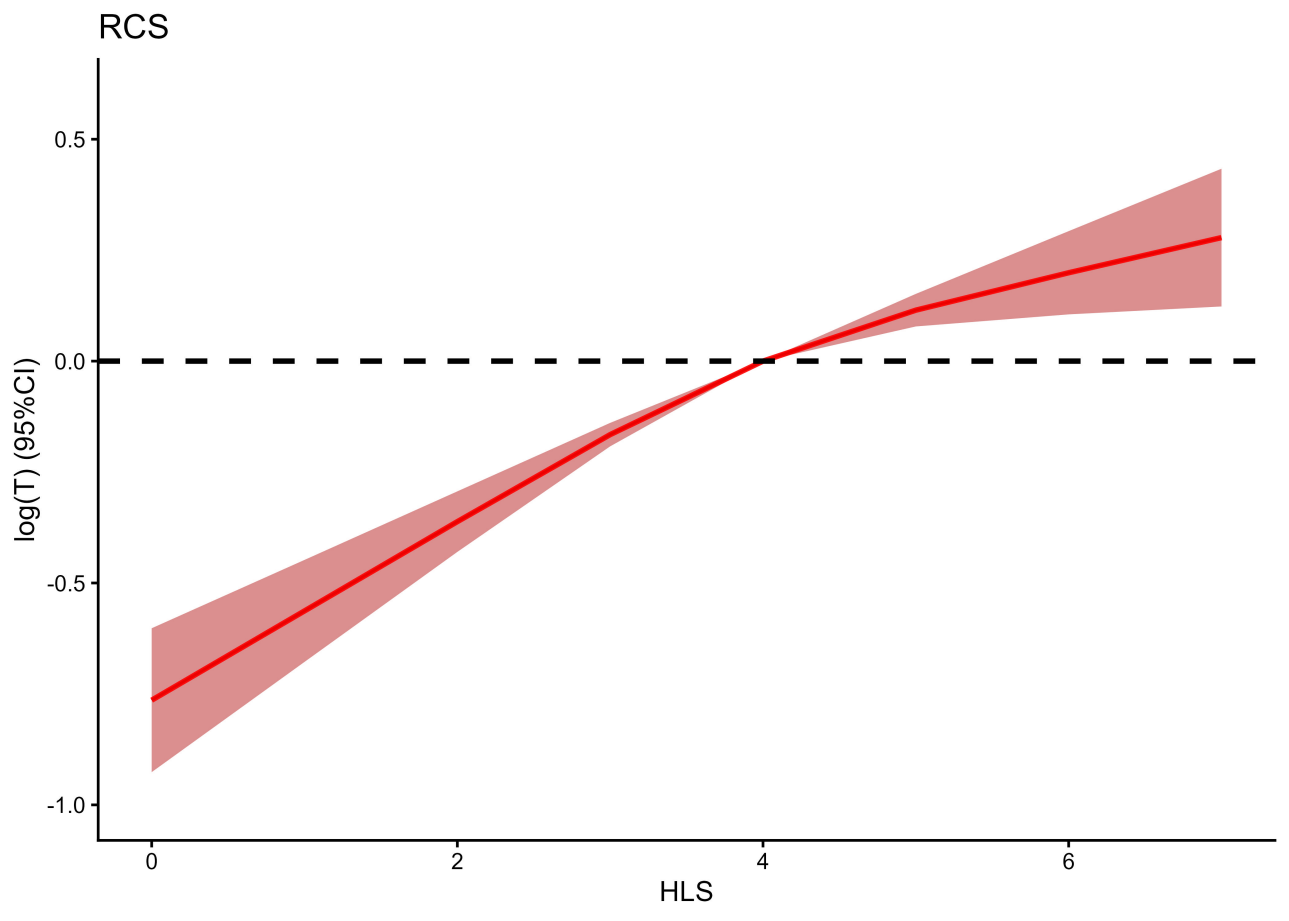
**Restricted cubic spline models for the relationship between HLS and log-transformation of survival time of developing depression (time to incident depression)**. The x-axis shows the HLS (range 0–7). The y-axis shows the log-transformed survival time (from accelerated-failure-time models), where higher values indicate a longer time to depression onset. Solid line represents the estimated association and the shaded area denotes the 95% confidence interval. The model was adjusted for covariates included in Model 3. RCS, restricted cubic spline.

In the restricted cubic spline analysis, the Wald test indicated a slight departure from linearity for HLS (χ^2^ = 4.33, *p* = 0.0365). As the HLS increased from 0 to 4, the log-transformation survival time showed a marked upward trend, although it remained below zero. Beyond an HLS of approximately 4, the log-transformation of survival time rose above zero, indicating a longer time to depression onset, and continued to increase, though at a less steep slope compared to the 0–4 score range (Fig. 2).

Over a follow-up of 2,434,775 person-years (median 13.2 years; maximum 15.3 years), 8400 (5.06%) deaths, 450 (0.27%) losses to follow-up, and 5507 (3.32%) incident depression cases were recorded. Among the depression patients, 103 (1.87%) were previously diagnosed with stroke, with a mean time from stroke to depression diagnosis of 2.67 (SD = 2.38) years. In the fully adjusted Model 3, participants with an HLS of 6-7 experienced a significantly longer time to depression onset than did those with an HLS of 0-2 (time ratio 1.81; 95% CI, 1.60–2.06; *p* < 0.001). Each 1-point increase in HLS was associated with an approximately 16.2% prolongation in the time to incident depression (time ratio, 1.16; 95% CI: 1.13–1.19; *p* < 0.001). Detailed results were shown in Table 2.

**Table 2.** **Time ratios for incident depression according to healthy lifestyle score in the UK Biobank**.

HLS	Model 1				Model 2				Model 3			
	Time ratio	LCI	UCI	*p*	Time ratio	LCI	UCI	*p*	Time ratio	LCI	UCI	*p*
0–2	Ref.				Ref.				Ref.			
3	1.25	1.15	1.36	<0.001	1.24	1.14	1.35	<0.001	1.23	1.13	1.35	<0.001
4	1.48	1.36	1.60	<0.001	1.45	1.34	1.57	<0.001	1.48	1.36	1.62	<0.001
5	1.69	1.55	1.85	<0.001	1.66	1.51	1.81	<0.001	1.66	1.51	1.83	<0.001
6–7	1.86	1.65	2.09	<0.001	1.80	1.60	2.02	<0.001	1.81	1.60	2.06	<0.001

Abbreviations: LCI, lower confidence interval; UCI, upper confidence interval.

### 3.3 Subgroup Analysis

When stratified by the occurrence of incident stroke, the association remained directionally consistent but differed in magnitude. Among participants without incident stroke, time ratios were comparable to those observed in the overall population, indicating a strong and graded delay in depression onset with increasing HLS (Table 3). In contrast, among participants who experienced a stroke before depression onset, the protective association of HLS was attenuated, with lower time ratios observed across all HLS categories (e.g., time ratio for HLS = 6–7: 1.47, 95% CI: 1.37–1.57) (Table 4). Nevertheless, all associations remained statistically significant (all *p* < 0.001), suggesting that a healthy lifestyle continues to confer benefits for delaying depression even in the presence of an incident stroke.

**Table 3.** **Time ratios for incident depression excluding incident stroke according to healthy lifestyle score in the UK Biobank**.

HLS	Model 1				Model 2				Model 3			
	Time ratio	LCI	UCI	*p*	Time ratio	LCI	UCI	*p*	Time ratio	LCI	UCI	*p*
0–2	Ref.				Ref.				Ref.			
3	1.26	1.15	1.37	<0.001	1.24	1.14	1.35	<0.001	1.23	1.13	1.35	<0.001
4	1.48	1.36	1.60	<0.001	1.45	1.33	1.58	<0.001	1.49	1.36	1.63	<0.001
5	1.70	1.55	1.86	<0.001	1.66	1.52	1.82	<0.001	1.67	1.51	1.84	<0.001
6–7	1.87	1.66	2.11	<0.001	1.81	1.61	2.04	<0.001	1.83	1.61	2.08	<0.001

**Table 4.** **Risk of incident depression including incident stroke according to healthy lifestyle score in the UK Biobank**.

HLS	Model 1				Model 2				Model 3			
	Time ratio	LCI	UCI	*p*	Time ratio	LCI	UCI	*p*	Time ratio	LCI	UCI	*p*
0–2	Ref.				Ref.				Ref.			
3	1.14	1.09	1.20	<0.001	1.14	1.08	1.19	<0.001	1.14	1.08	1.20	<0.001
4	1.26	1.20	1.32	<0.001	1.24	1.19	1.30	<0.001	1.25	1.19	1.32	<0.001
5	1.39	1.32	1.46	<0.001	1.36	1.29	1.43	<0.001	1.38	1.31	1.45	<0.001
6–7	1.49	1.39	1.59	<0.001	1.45	1.36	1.55	<0.001	1.47	1.37	1.57	<0.001

## 4. Discussion

In this large prospective cohort study, we observed that (1) a higher HLS was associated with a longer time to depression onset; and (2) this association was evident both among participants with and without incident stroke, although the magnitude of the association differed between these subgroups. In subgroup analyses stratified by incident stroke, higher HLS levels were consistently associated with delayed depression onset in both groups, but the time ratios were attenuated among participants who experienced a stroke prior to depression. These findings suggest that while stroke represents an important clinical context influencing depression risk, it does not substantially alter the overall protective association between a healthy lifestyle and depression.

Our study may indicate the critical importance of comprehensive lifestyle practices. Furthermore, we observed that a holistic healthy lifestyle plays a protective role against both the onset and progression of depression, providing supportive evidence to strengthen this area of research. Published evidence indicates that unhealthy lifestyles—such as poor diet, inadequate sleep, and physical inactivity—are associated with depression [21]. Conversely, combined interventions promoting exercise, healthy eating, sleep hygiene, and smoking cessation have been found to be efficacious in alleviating depressive symptoms and preventing relapse in patients with recurrent depression [22].

Although pharmacological treatments have demonstrated efficacy in improving outcomes for both stroke and depression, they often pose financial burdens and unavoidable systemic side effects, making them inaccessible or unsustainable for many families. In contrast, adherence to a healthy lifestyle is far more cost-effective and feasible to implement, with comprehensive benefits for overall health [23]. This positions lifestyle modification as a promising mainstream strategy for reducing stroke burden through primary prevention [23]. Importantly, this conceptual and public-health appeal is strongly supported by a growing body of epidemiological evidence. Epidemiological evidence consistently links modifiable lifestyle factors, such as moderate alcohol consumption [24], nonsmoking [25], physical activity [26], low adiposity [27] and healthy diets [28], to a reduced risk of stroke. Besides, both pharmacotherapy and psychotherapy for mental disorders exhibit constraints in their efficacy, especially in the context of acute symptom control and sustained management, frequently contributing to cases of treatment resistance [29]. In light of these limitations, contemporary clinical viewpoints are placing greater emphasis on the integration of healthy lifestyle practices—particularly regular physical activity and balanced nutrition—as a fundamental component of care [30,31,32].

The mechanisms through which healthy lifestyle factors influence mental disorders are likely complex and multifactorial. Some research suggests that consumption of “unhealthy” meals may elevate inflammatory markers and exacerbate depressive symptoms [33,34]. In recent years, the role of the gut microbiome in mental health has gained attention [35], with evidence indicating that the microbiome can be modulated by diet and exercise [36,37]. This represents one potential pathway linking health-related behaviors to mental disorders. Additionally, studies have shown that sleep restriction and low physical activity can lead to dysregulation of the hypothalamic-pituitary-adrenal (HPA) axis [38]. In the current study, positive social activities (e.g., interacting with friends, participating in group events, or attending courses) were considered. These activities are beneficial for general and psychological health, partly due to their link with increased physical activity [39]. Further investigation is needed to elucidate the underlying mechanisms by which different lifestyle factors affect mental health.

The attenuated associations observed among participants with incident stroke may reflect the substantial neurobiological and psychosocial burden imposed by cerebrovascular events, which could partially overwhelm the protective effects of lifestyle factors. The biological mechanisms linking stroke to depression involve shared pathophysiological pathways, including inflammation, oxidative stress, and endothelial dysfunction. Stroke acts as a traumatic event in the brain, triggering a robust inflammatory response in the affected regions. Cytokines released from damaged neurons can activate microglia, leading to widespread brain inflammation and ultimately contributing to depression [40]. Adherence to a balanced dietary pattern, such as the Mediterranean diet, which is considered “less inflammation-prone”, could mitigate such inflammatory conditions. Additionally, regular physical activity may reduce the risk of hypertension, the crucial modifiable risk factor for stroke [41], through several mechanisms, including improved lipid metabolism (increased HDL, lowered LDL), enhanced endothelial function, and reduced blood viscosity [42,43,44]. Stroke-related brain injury, functional impairment, and post-stroke inflammation are well-established contributors to depression risk, potentially limiting the extent to which lifestyle behaviors can delay depression onset in this population. Nevertheless, the persistence of a significant association suggests that lifestyle factors remain relevant even in high-risk clinical contexts.

### 4.1 Limitations

Several limitations should also be noted. First, lifestyle factors were measured at baseline (2006–2010) and may not have reflected previous lifestyle patterns. Potential changes in lifestyle after baseline assessment may have influenced our estimates. Second, certain lifestyle variables associated with incident depression, such as meal timing and exposure to nature [45,46], were not included in our analysis. Third, participants in the UK Biobank tended to be more health-conscious than the general population, which may have introduced selection bias and led to underestimated incidence rates of stroke and depression [47]. Moreover, the inclusion criterion of complete data on all healthy lifestyle factors may have further selected for individuals who were more health-aware, adhered to healthier behaviors, and possessed clearer cognitive function, thereby amplifying this selection bias. Last, diagnostic delay and under-ascertainment were possible because depression was identified from hospital records, which may have missed milder cases managed in primary care or diagnosed later in the recovery trajectory.

### 4.2 Future Directions

Building on our findings, several directions merit further investigation. First, repeated assessments of lifestyle over time may better capture behavioral changes and reduce exposure misclassification. Second, more comprehensive depression ascertainment—combining symptom scales, primary care records, and medication data—may improve outcome sensitivity and clarify heterogeneity in depressive phenotypes. Finally, given that the association between healthy lifestyle and depression appears largely preserved regardless of stroke occurrence, future research should investigate alternative biological and psychosocial pathways—such as inflammation and immune dysregulation, metabolic dysfunction, sleep disturbance, and social disconnection—that may underlie this association.

## 5. Conclusions

Overall, this large-scale prospective cohort study demonstrated that greater compliance with a combination of beneficial lifestyle behaviors was significantly associated with lower depression risk. Each incremental improvement in the HLS was associated with an approximately 16.2% longer time-to-depression onset. In both groups with and without incident stroke, patients with higher HLS were correlated with delayed depression onset. Although the association was weaker among those who experienced stroke, the overall protective effect of HLS on depression persists irrespective of incident stroke.

## Data Availability

The data that support the findings of this study are available from UK Biobank (http://www.ukbiobank.ac.uk/) but restrictions apply to the availability of these data, which were used under license for the current study, and so are not publicly available. Data are however available from the corresponding author upon reasonable request and with permission of UK Biobank.
